# The role of family size, employment and education of parents in the prevalence of intestinal parasitic infections in school children in Accra

**DOI:** 10.1371/journal.pone.0192303

**Published:** 2018-02-07

**Authors:** Akua Obeng Forson, Isaac Arthur, Patrick F. Ayeh-Kumi

**Affiliations:** 1 Department of Medical Laboratory Science, School of Biomedical and Allied Health Sciences, College of Health Sciences, University of Ghana, Legon, Accra, Ghana; 2 Department of Medical Microbiology, School of Biomedical and Allied Health Sciences, College of Health Sciences, University of Ghana, Legon, Accra, Ghana; Food and Drug Administration, UNITED STATES

## Abstract

**Introduction:**

Intestinal parasitic infections (IPIs) in school children are a public health problem in most developing countries.

**Methods and principal findings:**

A cross sectional survey was conducted from May to July 2016 with school children living in overcrowded urban slums in Accra, Ghana. A simple random sample of 300 children aged 2–9 years was collected. The study used structured pre-tested questionnaire and stool tests to obtain information on epidemiological, sanitation habits, employment and education status of parents and children. Data were analysed using appropriate descriptive, univariate and multivariable logistic tools of analyses. The mean age of participants was 6.9 years and 49% were males and 51.3% were females. *Giardia lamblia* was found in males (10.95%) and females (7.79%). Very low prevalences for *Schistosoma mansoni*, *Ascaris lumbricoides*, *Taenia* species, and *Entamoeba coli* were detected. Whilst children from mothers (62.2%) and fathers (55.6%) with no education were often infected, a few children from fathers (22.2%) and mothers (6.7%) with no jobs were infected. Most of the infected children’s (93.7%) parents did not have any knowledge of IPIs. The educational and employment status of the mothers [p = 1.0 and p = 0.422] was not significant, however, the family size was a predisposing factor (p = 0.031) for parasitic infections.

**Conclusions:**

Intestinal parasites were prevalent in children from overcrowded families and with no knowledge of IPIs. Educative programmes on IPIs, improving hygiene, and application of supportive programmes to elevate socioeconomic conditions may help reduce the burden of intestinal parasite carriage in children.

## Introduction

Intestinal parasitic infections (IPIs) are globally endemic and constitute one of the greatest causes of illness in developing countries [1]. IPIs can be transmitted directly (hand-to-hand contact) or indirectly (contact with contaminated food or water and from environmental surfaces) [2, 3] Varying climatic conditions, economic, cultural and sanitation practices are main contributing factors to the prevalence of intestinal parasites [3, 4]. People of all ages can be affected by IPIs, with most school children being more at risk. The helminths *Ascaris lumbricoides*, *Trichuris trichiura*, hookworms, *Hymenolepis nana*, and the protozoan *Giardia duodenalis*/*Giardia intestinalis* are the common intestinal parasites parasitizing more than 1 billion people worldwide [2–4]. In the last two decades, different studies have reported varying prevalences of IPIs among school children in Cuba (45–58%) [5], Pakistan (52.8%) [6], Mexico (65%) [7], Ethiopia (28% -95%) [8–11], Sudan (90%) [12], and Burkina Faso (84%) [13] to be associated with personal hygiene, socio economic status and educational levels of the parents.

The presence of IPIs can affect the mental and physical developments of children hence countries like Thailand, Uganda, Guatemala and Egypt have undertaken different programmes on nutrition, immunization and de-worming to promote health by influencing behaviour of parents towards preventing parasitic infections [14–17]. In Ghana, the Volunteer Partnerships for West Africa (VPWA) has supported the administration of de-wormers to some school children [18]. However, its impact has not been evaluated and the programme did not train or give guidance to parents and children on how to solve common health problems. Although previous studies in Ghana have reported 6.8% for *G*. *lamblia* and 5% for IPIs in children attending hospitals in the Greater Accra and Northern regions of Ghana, limited information is available on presumably healthy children living in urban slums in Accra, Ghana. This research was carried out to investigate the prevalence of intestinal parasites and the impact of socioeconomic factors, occupation, family size and education of parents on the transmission of IPIs among school children from 2–9 years of age residing in urban slums of Greater Accra.

## Methods

### Study location

Accra Metropolitan Area (AMA) is the capital of the Greater Accra Region of Ghana [19]. The city of Accra lies in the dry equatorial climatic zone and covers a total land area of 139.674 Km^2^. The Metropolitan Area shares common boundaries with La-dade kotokpon municipal from the east and Ga west municipal, Ga central municipal and Ga south municipal assembly from the west [20]. Some of the suburbs include Agbloblogshie, Timber market, Konkonba, Palledium, Jamestown, Adendeinkpo, Korle-gorno and Old Fadama (a slum called Sodom and Gomorah that inhabits more than 20,000 people) [21]. The condition of services, planning and environment in the suburbs is very poor with high housing density, poor road network as well as lack of good drinking water and hygienic sanitation.

### Subjects selection

Randomly, students attending 8 schools in Accra Metropolitan Area from March 2016 to July 2016 were included in this study after an informed consent was obtained from their parents because they were within 2–9 years of age. The consent forms were given to interested parents of the students to explain the objectives and procedures of the study. In addition, a self-administered questionnaire form was also given to the parents (see S1 File for Copy of Questionnaire on Socio-Demographic and Sanitary Facilities of Students).

The questionnaire sought to obtain information related to socio-demographic profile, environmental factors, and sanitary habits. School teachers assisted with the guidance of parents on how to fill the forms in cases where both parents were illiteracy.

### Data collection

Upon receipt of signed consent forms from parents, students were given stool containers with instructions to give to their parents to assist with the stool collection. A research assistant was responsible for the collection of stool specimens together with the questionnaire early in the morning. Stool specimens were collected from each participant and labelled before being transported to the laboratory within three hours after collection without any preservatives. Ice boxes were used to keep the stool samples during transportation.

### Analysis of stool samples/management

In the laboratory, stool samples were examined macroscopically for colour, consistency, presence of blood, mucus, pus and large worms [22]. This was done to obtain information on the type of parasitic infections that might be present. Consistency of the stool samples were also checked to determine whether there was any diarrheic stools or stools with unusual consistency were present. Stool samples were then fixed with 10% formalin. The parasites were examined by direct wet mount and formol-ether concentration. Briefly, for the direct wet mount, a drop of the emulsified stool samples was transferred onto both ends of a glass slide, a drop of Lugol’s iodine was added to one drop, leaving the other sample drop unstained. They were covered with a cover slip and examined first by 10x objective lens, and then by 40x for detailed identification of intestinal parasites [21].

The remaining stools was formol ether concentrated by emulsifying approximately 1 gm with 3 ml of 10% formol-saline in a test tube (21). The emulsified samples was poured into layers of gauze into another test tube and then 4 mls of diethyl ether was added to the filtrate from the stools. An additional 3 mls of 10% formol-saline was added to the filtrates to reach the 10mls mark and this was mixed by inverting and shaking intermittently for 1 min. The preparation was then centrifuged at 5000 x g for 5 mins. After centrifugation, the supernatant containing the debris, ether and formol saline was discarded and the sediments containing the parasites was re-suspended in 1ml formol saline. Four slides were prepared for each concentrated sample. Two of the slides were prepared and one slide was observed directly unstained and the other stained with iodine. The other two slides were stained with giemsa for the detection of coccidian parasites. Slides were observed with both low (X10, X40) and high (X100) magnifications.

### Study size

In order to achieve a 95% confidence level, the minimum study size was determined using the formula n = (z2 × p(1 − p))/e2 where n was the sample size, z was the standard score of 95% confidence interval, p was the prevalence (since no previous data existed, 50% was used) and e was the margin of error (1.96) with significance level set at p = 0.05.

### Data handling and statistical analysis

The data were entered into Microsoft Excel and analyzed using GraphPad Prism software, version 6. In all cases, P-values less than 0.05 were considered statistically significant. Initially the association between each exposure and the presence of infection was assessed using the Chi-squared test. Chi-square analysis was carried out to test for significance between prevalence of intestinal parasitic infections and risk factors for prevalence of intestinal parasitic infections. Odds ratios were computed to measure the strength of association. To determine independent risk factors for infection, logistic regression analysis was employed where appropriate.

### Ethical approval

The study was approved by the Ethics Committee of the School of Biomedical and Allied Health Sciences, University of Ghana. Ethics Identification Number: SAHS-ET/10339006/AA/MLS/2015–2016. Participation was voluntary and written consent was taken in accordance with the ethical committee’s guidelines. Permission was sought from Ghana Education Service (G.E.S), headmasters and class teachers before the samples were taken.

### Analysis

Descriptive statistics were expressed as mean and standard deviation (SD) in the case of age, years of education, crowding index and family monthly income. Categorical data were expressed in proportions and percentages for gender, parasites, employment and occupational status of parents. Chi square tests for proportion were applied for comparisons. The association between predictor variables (independent variables) with infection (dependent variable) was determined by odds ratios and their 95% confidence intervals.

## Results

### Prevalence of intestinal parasitic infections

In all, 45 (15%) (95% CI: 1.737; p = 0.1098) school children were infected with one or two intestinal parasites (Table 1). Thirty one (10.3%) and 14 (47%) children were infected with protozoans and helminths intestinal parasites respectively. The pathogenic *Giardia lamblia* [9.3% (28)] was the most prevalent (Table 1). *Ascaris lumbricoides*, *Taenia* species, *Hymenolepis nana*, *Strongyloides stercoralis*, *Schistosoma mansoni* and *Entamoeba coli* were not as prevalent (Table 1). *G*. *lamblia* was found in both males [16 (10.95%)] and females [12 (7.79%)]. Prevalence of infection was not statistically different by gender (p = 0.0318), however, those with multiple infections constituted 1.3% with 2 children (1 male and 1 female) being infected with *G*. *lamblia* and *H*. *nana* (Table 1).

**Table 1:** Prevalence of intestinal parasitic infections among the school children.

		Sexes		
Intestinal parasites		No. of Males (%) (n = 146)	No. of Females. (%) (n = 154)	Total no. (%) (n = 300)
**Single infection**				
**Protozoans**	*Giardia lamblia*	16 (10.95)	12 (7.79)	28
	*Entamoeba coli*	2 (1.36)	0	2
	**Total**	**18 (12.3)**	**12 (7.79)**	**30 (10)**
**helminths**	**Flukes**			
	*Schistsoma mansoni*	2 (1.37)	2 (1.3)	4
	**Tape worms**			
	*Taenia* species	2 (1.37)	0	2
	*Hymenolepis nana*	1 (0.68)	0	1
	**Nematodes**			
	*Ascaris lumbricoides*	2 (1.37)	1 (0.65)	3
	*Strongyloides stercoralis*	0	1 (0.65)	1
	**Total**	**7 (4.79)**	**4 (2.6)**	**11 (3.67)**
**Mixed (double) infections**				
	*Giardia lamblia & Hymenolepis nana*	1	1	2
	*Entamoeba coli & Giardia lamblia*	1	0	1
	*Gardia lamblia & Schistsoma mansoni*	1	0	1
	**Total parasites detected (%)**	**28 (19.18)**	**17 (11.0)**	**45 (15)**

### Descriptive characteristics and correlation of intestinal parasitic infections (IPIs)

The mean (±SD) age of the children was 6.96 ± 1.7 years. Among the different ages, infections were common in age 5(25%), 6 (23.3%), 3 (20%), and 7(17.8) (Table 2). Whilst ages 2 year and 4 years had no IPIs, low prevalences were found in ages 8 (11%) and 9 (8.6%).

**Table 2:** Univariate analysis of parents’ education and occupation with intestinal parasitic infection among children 2 to 9 years of age, residing in urban slums in Accra, Ghana who provided a stool sample for this study.

Variables		Total no. (%) (n = 300)	Infected (%) (n = 45)	Not Infected (%) (n = 255)	Odds ratio (95% CI)	P values
**Father’s education**	None	128 (42.7)	25 (55.6)	103 (40.4)	1.375 (0.8016–2.360)	0.154
	Basic	103 (34.3)	10 (22.2)	93 (36.5)	0.6093 (0.2950–1.259)	0.1164
	Secondary	41 (13.7)	5 (11.1)	36 (14.1)	0.7870 (0.2931–2.114)	0.4211
	< Secondary	28 (9.3)	5 (11.1)	23 (9.0)	1.232 (0.4451–3.409)	0.4285
**Father’s occupation**	None	51 (17)	10 (22.2)	41 (16.1)	1.382 (0.6460–2.957)	0.2583
	Unskilled labour	46 (15.3)	7 (15.6)	39 (15.1)	1.017 (0.4283–2.415)	0.5576
	Skilled labour	73 (24.3)	11 (24.4)	62 (24.3)	1.005 (0.4916–2.056)	0.5565
	Market Traders	106 (35.3)	16 (35.6)	90 (35.3)	1.007 (0.5424–1.871)	0.5466
	Professional	24 (8)	1 (2.2)	23 (9.0)	0.2464(0.03244 t- 1.871)	0.1164
**Mother’s education**	None	188 (62.7)	28 (62.2)	160 (62.7)	0.9917(0.5945–1.654)	0.5419
	Basic	85 (28.3)	15 (33.3)	70 (27.5)	1.214 (0.6393–2.306)	0.3292
	Secondary	19 (6.3)	2 (4.4)	17 (6.7)	1.250(0.2723–5.739)	0.5092
	< Secondary	8 (2.6)	0	8 (3.1)	0.3303 (0.01872–5.827)	0.2782
**Mother’s occupation**	None	13 (4.3)	3 (6.7)	10 (3.9)	1.7 (0.4501–6.421)	0.3198
	Unskilled labour	17 (5.7)	2 (4.4)	15 (5.9)	0.7556 (0.1670–3.418)	0.5251
	Skilled labour	38 (12.7)	3 (6.7)	35 (13.7)	0.4857 (0.1432–1.647)	0.1761
	Trader	216 (72)	36 (80.7)	180 (70.6)	1.111 (0.7433–1.661)	0.3466
	Professional	16 (5.3)	1 (2.2)	15 (5.9)	0.3778 (0.04866 to 2.933)	0.2932

Out of the 45 infected, children from mothers who were traders (80%) were more positive than children whose mothers had professional jobs (2.2%). Whilst children from fathers who were involved in trading (35.6%) and skilled labour (24.4%) were often infected, few children from fathers with professional jobs (2.2%) were infected. The educational levels of the parents were found to vary, however, children from mothers [28 (62.2%)] and fathers [25 (55.6%)] with no education were often infected (Table 2).

Status of occupation and education levels of the mothers (OR = 1.750, p = 0.422 and OR = 0.9779, p = 1.0) and fathers (OR = 1.491, p = 0.3879 and OR = 1.845, p = 0.0719) did not represent a risk of infection for the children (Table 3). However, the family sizes of the children was a predisposing factor for parasitic infection (OR = 0.4750, p = 0.031, Table 3). Furthermore, most of the parents reported their children always had clean cut nails (68.3%), however most of those children were found to be infected (73.3%) with IPIs compared to those who always did not (32.5%). Whilst children whose parents had no knowledge (93.7%) of intestinal parasites were often infected than those who had knowledge (6.3%) of parasitic infections, no significant difference (p = 0.5168) was found for IPIs.

**Table 3:** Bivariate analysis for factors potentially associated with Intestinal parasites among children 2 to 9 years of age, residing in urban slums in Accra, Ghana who provided a stool sample for this study.

Variables		Positive (%) (n = 45)	Negative(%) (n = 255)	Odds ratio (95% CI)	P value
**Sex**	Males	28 (62.2)	118 (46.3)		
	Females	17 (37.8)	137 (53.7)		
				1.912 (0.9972 to 3.667)	0.0534
**Father’s education**	None	25 (55.6)	103 (40.4)		
	Basic school and Above	20 (44.4)	152 (59.6)		
				1.845(0.9735 to 3.495)	0.0719
**Father’s occupation**	None	10 (22.2)	41 (16.1)		
	Working	35 (77.8)	224 (83.9)		
				1.491 (0.6847 to 3.248)	0.3879
**Mother’s education**	None	28 (62.2)	160 (62.7)		
	Basic school and Above	17 (37.8)	95 (37.3)		
				0.9779 (0.5085 to 1.881)	1.0
**Mother’s occupation**	None	3 (6.7)	10 (3.9)		
	Working	42 (93.3)	245 (96.1)		
				1.750 (0.4622 to 6.626)	0.422
**Family numbers**	≥4	20 (44.4)	160 (62.7)		
	≤ 3	25 (55.6)	95 (37.3)		
				0.4750 (0.2503–0.9014)	0.031**
**Clean and cut finger nails**	Yes	33 (73.3)	172 (67.5)		
	No	12 (26.7)	83 (32.5)		
				1.327 (0.6518–2.702)	0.4904
**knowledge of intestinal parasites**	Yes	4 (8.9)	16 (6.3)		
	No	41 (91.1)	239 (93.7)		
				1.457 (0.4638–4.579)	0.5168

## Discussion

In the present study, the prevalence of intestinal parasitic infections and their relationship with socio-demographic, family size, knowledge and occupation of parents of school children living in an urban slum in Accra, Ghana was studied. Previous studies have tackled intestinal parasitism in Ghanaian children [23, 24]; however, this is the first study to investigate influencing variables of parents and family sizes with paediatric parasitic infections in an urban slum in Accra, Ghana. The present study revealed 15% of primary school children, aged 2–9 years were infected by one or more intestinal parasites. Our findings are similar to Shakya *et al*., [25]) study from Nepal which reported a 14% prevalence. Compared to our findings, the prevalence of intestinal parasitic infections among the children was significantly lower than 85% reported by Osman *et al*., [26] and the 52.8% by Merhaj *et al*., [6] in children in Lebanon and Pakistan. The differences in prevalences in the countries may be associated with varying pathogenicity or asymptomatic carriage of the parasites and sampling methods. Young school going children have been reported to be at higher risk for intestinal parasitic infections (IPIs) [27, 28]. This study identified children aged 5 (13%), 6 (22.2%), and 7 (28.9%) years as the most infected with IPIs. Although our findings are similar to Tandukar *et al*., [29] study, the reasons for the increase in IPIs with age could be due to the fact that as children grow older, the exposure to IPIs increases, but further exploration through prospective studies with a bigger sample size is required.

In this study *Giardia lamblia* and *Entamoeba coli* were the common protozoan identified from the stool samples of 30 (10%) children. Whilst varying prevalence for *Ascaris lumbricoides*, *Schistsoma mansoni*, *Taenia* species, *Hymenolepis nana*, *Strongyloides stercolaris* were detected in 11 (3.7%) children, a few [4 (1.3%)] children had mixed infection with *G*. *lamblia* and *H*. *nana*, or *E*. *coli* and *S*. *mansoni* (Table 1). The prevalence of protozoan parasites detected are lower than reports from Cuba [5], Nepal [25], Pakistan [6] and Ethiopia [30]. Furthermore, in conformity to Mehraj *et al*., [6] and Ahmed *et al*., [31] representing studies from other urban localities in Pakistan, no hookworm was detected in this study. Although a previous study by Reither *et al*., [24] with children from northern Ghana which has rural characteristics (with hamlets and thatched mud-wall huts scattered over a vast area) identified hookworm, it is obvious that parasitic profile of urban slums is different from rural areas and further studies are required to make substantive conclusions. Transmission of the detected protozoan and helminths parasites have been previously reported to be associated with poor sanitation and contaminated water [5, 6, 24]. Our diagnostic sensitivity may have been improved if we had taken three consecutive stool samples, but this was not done because the children and their parents were not willing to co-operate.

Low socioeconomic status (SES) has been reported to be a risk factor for IPIs [32]. The effect of SES on risk of parasitic infections in particular is complex in nature and could be attributed to sources of drinking water and food, hygiene, environment, access to education, and living conditions of individuals. We took a combination of education [(maternal p = 1.0, OR = 0.9779) (paternal p = 0.3879, OR = 1.912)] and occupation [(maternal p = 0.422, OR = 1.750) (paternal p = 0.0534, OR = 1.491)] of the parents as a proxy measure of SES in this study, but we found they were not associated with IPIs in the school children.

However, in agreement with Shakya *et al*., [25], and Quihui *et al*., [7] studies from Nepal, and Mexico, children of illiterate and basic education fathers [(55.6%), and (22.2%)] and mothers [(62.2%) and (33.3%)] were often infected than the literate ones. This has revealed that the better educated the parents are, the lower the prevalence of IPIs in children as previously reported by Nematian *et al*., [32] and Wamani *et al*., [15] with children from Iran and Uganda. The differences in prevalences in the different countries may be due to varying social economic conditions and environmental behaviours in the different countries.

In addition, mothers who were traders many infected children (80.7%) compared to children (35.5%) from fathers who were traders. This could be that as mothers who are often the main care givers are busy with their trading activities, there is an increase in exposure to IPIs of school aged children in urban slums in Accra. Further exploration is required to link trading activities of mothers as an independent risk factor for IPIs.

Furthermore, children with no knowledge of intestinal parasitic infection [41 (14.6%)] were often infected with intestinal parasites. Persuasive education on IPIs, improving hygiene, and application of supportive programmes for parents to elevate socioeconomic conditions may assist in the reduction of parasitic infection in children.

Personal hygiene has been described to have an impact on parasitism [33]. Although children whose parents claimed to always have clean cut nails were often infected (16.1%), children from parents with more children (20.8%) were often infected. In agreement with Maia *et al*., [34] study from Brazil, the occurrence of IPIs was statistically associated with children with more household members (OR = 0.4750, p = 0.031). Overcrowded conditions of household members can lead to intra-family transmission from close contacts of crowded houses.

## Conclusion

In conclusion, although intestinal parasitosis among the school children was found to be 15%, IPIs are important public health problem globally. Preventive programmes on awareness of the infectious diseases, improving hygiene, and application of supportive programmes to elevate socioeconomic conditions may reduce the burden of parasitic infection. In addition there is a need for continuous mass scale deworming programmes to enable eradication of IPIs and studies on parasitic infections in different population settings are required to help develop effective prevention and control strategies for the eradication of IPIs in children.

## Limitations

The current study had certain limitations; only one stool sample was examined instead of the ideal three consecutive samples due to poor cooperation of parents. The results of this study cannot be generalized to all Ghanaian school children as the variation of the included variable may vary in different seasons, or among ethnic groups in their geographic and socio-economic settings in Ghana. Other limitations include lack of baseline data on if the students have ever been infected or treated for parasitic infections.

## Supporting information

S1 File Copy of questionnaire on socio-demographic and sanitary facilities of students. (DOCX)
