# Supplementary material for: The role of family size, employment and education of parents in the prevalence of intestinal parasitic infections in school children in Accra
Source: PLoS One. 2018 Feb 7;13(2):e0192303. doi: 10.1371/journal.pone.0192303 (PMC5802905; doi:10.1371/journal.pone.0192303)
Supplement: S1 File — (DOCX) [file pone.0192303.s001.docx]

1 Supporting Information

**Copy of questionnaire on Socio-Demographic and Sanitary Facilities of Students**

NAME OF PARTICIPANT (optional)…………………………………………………………

1. Age….. [………….]

2. Gender….. [Male], [Female]

3. Place of stay. (Residence)….. [………………………………………………….]

4. Ethnicity…….. [……………………………………………………………..]

5. Family size [……] and by composition Males [………..] Female […………]

6. Source of Drinking Water **[A]** Sachet (pure) water

**[B]** Well (ground) water

**[C]** Tap (pipe) water

**[D]** Water from River.

8. Location of the school [……………………………………………………………………]

9. Number of students in class [………………………………………………………………..]

10. Known Health condition (for the past 3 weeks) [……………………………………………………]

(i) If any state [e.g vomiting, skin rashes, watery stool with blood, mucus etc.……………………………………………………………………………………………]

(ii) When was the last time you dewormed or took any antibiotic……………………………

(iii) Do you normally feel itchy at the anus……………………………………………………

11. The toilet facilities **[A]** Latrine **[B]** Water closet (W C) **[C]** In water bodies or bush.

**ANSWER [YES], [NO] OR [I DON’T KNOW] BY TICKING**

1. Do you wash hands after and before eating? [Yes], [No]

2. Do you wash hands after using the toilet? [Yes], [No]

3. Do you wash all fruits and vegetables before eating them? [Yes], [No]

4. Do you or your parent rear (domestic) animals in the house? [Yes], [No]

(i) What type of animal………………………………………………………………………

(ii) Do you play with domestic animals or pet ? [Yes] [No], [Not always]

5. Do you have pets in the house? [Yes], [No], [Not always]

(i)What type of pet (e.g Dog, Cat, Pig, Dove, etc.) …………………………………………….

6. Do you live in an area where people rear animals? [Yes], [No], [Not always]

(i)If yes, what animal? ………………………........................................................................

7. Do you always cut your fingernails? ........................................... [Yes], [No], [Not always]

How many times in a month? .............................................................................................

8. Do you wear shoes to school or when playing? [Yes], [No], [Not always]

9. Have you seen blood or worms in your stool before? [Yes], [No], [Not always]

10. Do you play with domestic animals or pet (specify if yes) [Yes] [No] [Not always]

11. Do you normally walk bear footed [Yes] [No] [Not always]

12. Do you go out to play after school? [Yes] [No] [Not always]

(i) What area do you go to play…………………………………………………………………

(ii) Are there any rubbish dumps or dirty gutters around area of play? ................................

13. Do you eat pork? [Yes] [No] [Not always]

14. Do you eat beef? [Yes] [No], [Not always]

15. Do you or your parents buy food from street food vendors? [Yes] [No], [Not always]

16. Do you know anything about intestinal parasites? [Yes] [No]

17. Has your parents been to school before? [Yes] [No]……

If yes specify which level….……………………………………………………………………

18. What work do your parents do? ............................................................................................
